# Challenges experienced by nurses in implementing Adolescent and Youth Friendly Services in clinics of the Limpopo province

**DOI:** 10.4102/hsag.v29i0.2472

**Published:** 2024-09-26

**Authors:** Anna M. Malahlela, Livhuwani Muthelo, Masenyani O. Mbombi, Tshepo A. Ntho, Thabo A. Phukubye, Peaceful N. Ntshayintshayi, Tendani A. Ramalata

**Affiliations:** 1 Department of Nursing Science, Faculty of Health Sciences, University of Limpopo, Polokwane, South Africa

**Keywords:** adolescent, Youth Friendly Services, challenges, nurses, implementation

## Abstract

**Background:**

Effective implementation of Adolescent and Youth Friendly Services (AYFS) that are accessible, acceptable and effective for diverse youth population groups is significant to enhancing youth health. Because of various factors, improving youth health is a challenge in clinics in rural areas.

**Aim:**

This study aims to explore and understand the challenges nurses experience while implementing AYFS programmes in Kganya local area clinics of the Capricorn district, Limpopo province, South Africa.

**Setting:**

The study was conducted in the clinics of Kganya local area of Capricorn district, Limpopo province, South Africa.

**Method:**

A qualitative, phenomenological, exploratory, descriptive design is employed in this study. Twelve nurses were selected using purposive sampling. Data were collected through semi-structured interviews with an interview guide. Notably, data were analysed using Tesch’s open coding method.

**Results:**

Two themes emerge from this study: ‘Challenges experienced related to the implementation of Adolescent and Youth Friendly Services’ and ‘Suggestions to improve Adolescent and Youth Friendly Services’.

**Conclusion:**

The results of this study confirm that nurses experience diverse challenges while implementing AYFS. Challenges include a lack of trained staff and material resources and the negative attitudes of adolescents, parents and community members.

**Contributions:**

This study’s findings might help identify the gaps in implementing the AYFS programmes. The findings may assist policymakers and the National Department of Health (NDoH) to monitor and review the effectiveness of the AYFS programme standards.

## Introduction

Adolescence and young adulthood are stages of rapid physical and psychosocial change and development and require proper management. These stages are characterised by less parental control and increased influence of peers and social media. Furthermore, these stages are often associated with a rise in experimentation and exploration, a search for identity and an increase in risky behaviour, alcohol and substance abuse, possible sexual and reproductive health problems, violence and mental illness (Cooper, De Lannoy & Rule 2015). Consistent with the demographic profile of adolescents in South Africa, individuals between the ages of 15 and 35 years are considered youth, while adolescence pertains explicitly to those aged 10–19 years (Maluleke 2018). Nevertheless, this study employs the age range of 10–24 years for the adolescent and youth population, as outlined in the African Adolescent and Youth Health Policy of 2017 (National Department of Health [NDoH] 2017).

Worldwide, 80% of 1.2 billion adolescents live in developing countries with inadequate access to health services such as sexual and reproductive health care (Beksinska et al. 2014). Because of social stigma, poor access to information and services and poor implementation of health policies, adolescents and youth face health challenges (Okeke et al. 2022). In South Africa, the NDoH introduced the Adolescent and Youth Friendly Services (AYFS) Policy in 2017; this aims to provide a practical and realistic approach to health programming. The Adolescent and Youth Health Policy seeks to change the perception of effective health promotion for adolescents and youth in South Africa (NDoH 2017; James et al. 2018). As a result, an AYFS programme was introduced in public health facilities by the National Department and partners to develop and implement standardised health programmes and services to enhance the health and well-being of young people aged between 10 and 24 years (NDoH 2017). According to Thomée et al. (2016), implementing AYFS will improve youth health as long as the services remain accessible, acceptable and effective for different youth population groups.

Globally, the World Health Organization (WHO 2015) aimed to assist policymakers and health service planners improve the quality of health care services through the formation of global health standards. Furthermore, the WHO intended to ensure that adolescents obtain the health services needed to promote, protect and improve their health and well-being. Sweden is one of the few countries that has integrated AYFS into the public health system; this is well sustained to meet young people’s needs (Thomée et al. 2016). Therefore, the Swedish youth clinics are regarded as role models of AYFS, because they provide services such as advice about and treatment of sexual problems to youth. This service includes people with disabilities and victims of sexual violence. In addition, the Swedish youth clinics offer services to young people at specific times; their free services include counselling on contraceptives, sexual intercourse and sexually transmissible disease prevention (Thomée et al. 2016). In sub-Saharan Africa, human immunodeficiency virus (HIV), sexually transmitted infections and unplanned pregnancies have been reported to be prevalent among adolescents and young adults (Smith et al. 2018). Thus, providing accessible AYFS is essential to reduce the health challenges young people experience, such as poor mental health, substance abuse, problems related to under- or over-nutrition, infectious diseases and sexual reproductive health (NDoH 2017). In 2016, South Africa had the highest rate of HIV infections among adolescents worldwide, accounting for nearly 18% of global infections among 15–24-year-olds (Mendelsohn et al. 2018).

In South Africa, the National Adolescent Friendly Clinic Initiative (NAFCI) programme was conceptualised and implemented by the Reproductive Health Research Unit (RHRU) between 1999 and 2005 (WHO 2009). National Adolescent Friendly Clinic Initiative aims to help the public sector respond to the needs of the youth appropriately, improve the quality of AYFS at the primary care level and strengthen the public sector’s ability. The NAFCI-initiated objectives are to provide accessible and good health care services to adolescents, standardise AYFS in nationwide clinics and capacitate health care workers to provide high-quality adolescent and youth health services (Smith et al. 2018). Baloyi (2006) conducted a study on implementing the NAFCI programme in Limpopo, South Africa. The findings revealed that adolescents utilise the programme’s services, particularly those concerning contraception, sexually transmitted infection prevention and pregnancy. However, Baloyi (2006) noted that, despite young people’s using NAFCI in the Limpopo province, teenage pregnancy and sexually transmitted infection rates remained a concern.

### Problem statement

Through her experience as a practice nurse, the primary author learned and observed that nurses encounter various challenges when implementing AYFS programmes in clinics. These obstacles are caused by a range of factors like inadequate infrastructure, a shortage of staff and inadequate training and equipment. The primary author’s observations are supported by monthly clinic peer reviews and the ideal clinic assessment, which highlights the non-performance and difficulties faced by nurses in the Kganya local area when implementing the AYFS programme. The South African AYFS initiative is one of the few projects that has been scaled to a national level (Geary et al. 2015). The availability of AYFS in health care facilities is crucial to support the well-being and stability of adolescents and youth.

Despite the various health challenges faced in South Africa, AYFS remains committed to implementing a standards-based approach to improve the quality of care provided to adolescents and youth (WHO 2012). From the previous discussion, one can see that there is a pressing need to mitigate the challenges experienced during the implementation of the AYFS to enhance the accessibility of health care services for adolescents and youth in the rural communities of Limpopo province. Thus, a clearer understanding of the challenges experienced by nurses in implementing the AYFS programme would assist nurses and stakeholders in enhancing the implementation process and would contribute to the realisation of Sustainable Development Goal (SDG) 3, which seeks to ensure healthy lives and well-being for all individuals at every stage of life. Policymakers and the NDoH gain guidance in continually assessing and evaluating the efficiency of all stages of the AYFS programme.

### Study aim

This study aims to understand the challenges nurses experience in implementing AYFS in clinics of the Kganya local area of Capricorn district, Limpopo province, South Africa.

#### Study objectives

This study aims:

- To explore and describe the challenges nurses experience when implementing AYFS in clinics of the Kganya local area of Capricorn district, Limpopo province, South Africa.
- To provide recommendations for the nurses to facilitate the implementation of AYFS programme in clinics of the Kganya local area of Capricorn district, Limpopo province, South Africa.

### Research methods and design

This study employs a qualitative, phenomenological, exploratory, descriptive design to understand the challenges nurses experience when implementing the AYFS programme in clinics of the Kganya local area of Capricorn district, Limpopo province, South Africa. In addition, the Consolidated Criteria for Reporting Qualitative Studies checklist was used to guarantee that every essential aspect of this qualitative manuscript was precisely incorporated (Tong, Sainsbury & Craig 2007).

### Population and sampling

A non-probability purposive sampling method was used in this study. At the time of the study, the population included 41 nurses working in accredited facilities to provide AYFS (Polit & Beck 2022). The local area manager of the Kganya local area assisted with identifying clinics with nurses who met the study inclusion criteria. These criteria include all nurses trained for AYFS from the Kganya local area, whose clinics have been accredited for providing the AYFS programme and were selected to participate in the study. All nurses outside the Kganya local area who were not involved in implementing AYFS were excluded from participating in this study. At the time of this study, 16 out of 41 nurses working in accredited clinics in the Kganya local area were trained in AYFS. However, the study reached the principle of data saturation after interviewing 12 of the 16 nurses who had indicated their willingness and availability to participate. According to Hennink and Kaiser (2022), saturation is a crucial indicator that the sample is adequate for the studied phenomenon and that the collected data adequately capture the diversity, depth and nuances of the studied issues, thus demonstrating confirmability.

### Study context

This study was conducted in the clinics of the Kganya local area of the Capricorn district, Limpopo province, South Africa. The Capricorn district is one of the five districts of the Limpopo province. The Kganya local area has six fixed clinics and one mobile clinic. The area serves communities from Mankweng, Ga-Mamabolo, Ga-Molepo and Ga-Mothapo. The clinics include J Mamabolo, Sehlale, Molepo, Mamotshwa, Phuti and E Lekganyane.

### Data collection

Data were collected through one-on-one, semi-structured, in-depth interviews between January and March 2021, in line with Kvale and Brinkman’s seven stages of qualitative interview investigations (Kvale & Brinkman 2015). The stages are thematising, designing, interviewing, transcribing, analysing, verifying and reporting. Interviews were conducted following an interview guide, with a series of questions to be asked of all participants. Probing and reflective techniques assisted the researcher in making follow-ups on interesting avenues that emerged during the interview sessions (Polit & Beck 2022). The probing and reflective technique was adopted from Russell Bernard’s Taxonomy of probing and includes silence, encouragement, elaboration, clarification, affirmation, reflective probing and recapitulation probing (Bernard 2013). A recruitment letter was given to the local area manager of Kganya to distribute among operational managers and nurses who had received training in AYFS. The letter explains the study details and invites interested AYFS-trained nurses to participate in the study. Subsequently, a 10-to-15-min slot was requested from the operational managers of each clinic on Wednesdays during shift changes; most nurses were present to recruit participants. All nurses interested in participating in the study voluntarily gave their names and contact details after a staff meeting, and the primary author provided them with informed consent forms. The primary author contacted nurses who obtained informed consent forms after 3 days; they agreed to participate in the study voluntarily. A convenient date and time were scheduled, and a signed informed consent form was obtained.

Interviews were conducted in private cubicles free from distractions and lasted between 35 and 60 min. All 16 trained AYFS nurses were recruited and expressed their willingness to participate in the study. However, the data collection process was concluded after interviewing the 12th participant, as data saturation had been reached. The interview guide was piloted on three nurses before the interviews; these nurses were excluded from the main study. With the participants’ written and verbal consent, the first author conducted the interviews and used a digital voice recorder to record them; field notes were also taken.

### Data analysis

Data were analysed using Tesch’s open-coding method of data analysis as described by Creswell and Creswell (2017). Tesch provides eight steps to consider when analysing qualitative data using a thematic approach:

#### Step 1: Reading through the data

Every audio-recorded interview was transcribed verbatim. The principal author made sense of the data by examining each verbatim transcript several times. Her understanding of the significance of the data segments was enhanced during this process. She read and assimilated the transcripts and noted the authors’ ideas. The independent coder received the transcribed data and used the same data analysis procedure used by the researchers.

#### Step 2: Reduction of the collected data

The author then took the collected data and condensed it into codes by analysing the frequency of concepts used in the verbatim transcriptions. She then identified similar topics that emerged during the data reduction process and grouped them together while clustering those without a clear association separately.

#### Step 3: Asking questions about the meaning of the collected data

The author questioned herself about the interview transcriptions, using the frequency-based codes of the concepts. Questions included ‘What is this about?’ and ‘What is the underlying meaning?’ The author grasped the meaning of the data and this led to coding.

#### Step 4: Abbreviation of topics to codes

Codes were assigned to the topics that the author abbreviated, and these codes were marked besides the corresponding parts of the transcription. All these codes were written in the margins of the transcripts.

#### Step 5: Development of themes and categories

Themes and categories were developed by the researcher using the coded data and associated texts. The list was then reduced by grouping related topics together to give meaning to the themes and categories.

#### Step 6: Compare the codes, topics and themes for duplication

The author verified that there was no repetition in the work. There was no necessity to refine the codes, topics and themes.

#### Step 7: Initial grouping of all themes and categories

All data of each theme were compiled into a single column. The authors carried out an initial analysis and then had a discussion with the independent coder to reconcile the themes and categories identified independently by everyone. A further meeting with an independent coder was held to establish a consensus on the themes and categories.

#### Step 8: Recoding

There was no need for recoding.

### Measures to ensure trustworthiness

This study utilised Guba and Lincoln’s (1985) four criteria of trustworthiness: credibility, transferability, dependability and conformability, as cited in Ramathuba and Ndou (2020). Credibility in this study was ensured through prolonged engagement in the interviews with participants, lasting from 35 to 60 min and the engagement with the transcribed data. Member checking was done after each interview, where the researcher summarised the information and confirmed its accuracy with the participant. Furthermore, **confirmability** in this study was ensured through the second and the third authors’ listening to the audio recordings to confirm the interpretation and conclusions of the first author. A meeting was held with these three authors to confirm the identified themes. A meeting with an independent coder and supervisor was held, and both worked autonomously to analyse and confirm their themes and categories, reaching a consensus. To ensure **dependability,** triangulation was utilised by conducting face-to-face interviews, using a digital voice recorder, taking field notes and allowing for effective auditing of the data collected. **Transferability** was established using the purposive sampling method to nominate nurses with knowledge of and experience in implementing the AYFS. The collection of data continued until data saturation was reached after interviewing 12 participants. Additionally, transferability was ensured through a thorough, thick description of the study’s context, participants and methodology.

### Ethical considerations

The researcher obtained ethical clearance from the University of Limpopo, Turfloop Ethics Research Committee (TREC/147/2020/PG). The Department of Health in Limpopo province also granted permission to conduct research in specific clinics. Furthermore, the operational managers of the clinic in Kganya local area of the Capricorn district, Limpopo province, South Africa, granted permission for the study. Participants were given written and verbal explanations of the study’s purpose and objectives before data collection. Participants were informed that interviews would be audio recorded, and field notes would be taken. Participants willingly participated in this study, having signed an informed consent form. They were informed that they could withdraw from participating in the study at any point without any penalty. Interviews were conducted in a private consulting room to ensure the participants’ privacy. Anonymity and confidentiality were ensured by assigning each participant a pseudonym (Participants 1, 2, 3, etc.) to ensure that no information obtained during the study would be linked to the participants. The study was conducted during the coronavirus disease 2019 (COVID-19) pandemic, and protocols were adhered to by both the primary author and the participants. Transcripts, field notes and audio recordings are kept secure for 5 years in a computer protected by a password; this computer is in a locked office to ensure privacy.

## Results

The results of this study are presented according to the demographic data (Table 1) and include the themes and subthemes (Table 2).

**TABLE 1:** Demographic profile of the participants.

Pseudonyms	Age in Years	Gender	Years of Experience in AYFS (years)
Participant 1	27	Female	0–2
Participant 2	47	Female	6 or more
Participant 3	57	Female	6 or more
Participant 4	50	Female	3–5
Participant 5	39	Female	3–5
Participant 6	32	Male	3–5
Participant 7	41	Female	3–5
Participant 8	35	Female	3–5
Participant 9	44	Male	3–5
Participant 10	36	Female	3–5
Participant 11	52	Female	6 or more
Participant 12	48	Male	3–5

AYFS, Adolescent and Youth Friendly Services.

**TABLE 2:** Themes and subthemes.

Themes	Subthemes
1. Challenges experienced related to the implementation of AYFS	1.1. Lack of adherence to AYFS schedule by youth because of distance and service times 1.2. Lack of commitment to offer AYFS by professionals at multiple levels 1.3. Lack of trained professionals to implement AYFS 1.4. Lack of infrastructure and material resources problematic for proper implementation of AYFS 1.5. Existing poor relationship between nurses and youth that affects the implementation of AYFS 1.6. Lack of funding to support AYFS as a possible contributor to the failure of the AYFS programme 1.7. Lack of monitoring and evaluation of AYFS impacts the failure of the programme 1.8. Negative attitudes of youth, parents and community members experienced by professional nurses towards AYFS.
2. Suggestions to improve AYFS	2.1. Appointment of relevant health professionals to implement AYFS 2.2. Training of health professionals to implement AYFS 2.3. Establishment of well-equipped infrastructure dedicated to AYFS 2.4. Provision of relevant and enough material resources for easy implementation of AYFS 2.5. Awareness campaigns and programmes about AYFS in communities for information giving 2.6. Applications and requests for funding to support AYFS and its sustainability

AFYS, Adolescent and Youth Friendly Services.

### Demographic data of participants

Table 1 indicates the demographic characteristics of the participants. Of the twelve participants, nine were female and three were male. Three participants were aged between 21 years and 35 years, seven participants were aged between 36 years and 50 years and two participants were aged 51 years and above. In terms of years of experience, eight participants had 3–5 years of experience, three participants had 6 or more years and only one nurse had 0–2 years of experience. The data are categorised based on pseudonyms, age in years, gender and years of experience in AYFS.

### Theme 1: Challenges experienced related to the implementation of Adolescent and Youth Friendly Services

During interviews, participants in this study were able to describe their views on challenges they experienced during the implementation of AYFS. Eight categories emerged from the challenges nurses experience when implementing AYFS in the clinics of Kganya local area, Capricorn district, Limpopo province, South Africa. The categories are discussed in the following sections.

#### Subtheme 1.1: Lack of adherence to Adolescent and Youth Friendly Services schedule by youth because of distance and service times

Signs displaying the time slots for the AYFS programme were posted on notice boards in various clinics of the Kganya local area of Capricorn district, Limpopo province, South Africa. It is important to note that AYFS was dedicated to the hours between 14:00 and 17:00, Monday to Friday. Although the youth knew of the scheduled times for AYFS, the study found that they did not follow the programme’s expected schedule. The following extracts from participants confirm this:

‘The only high school we manage to serve is nearby. I render Youth Friendly Service, especially to the school children in the afternoon, but unfortunately, I have challenges with the youth from far away villages since they come late, and the Youth Friendly Service is rendered from 2 p.m. to 5 p.m., and this prevents us from giving them full service.’ (Participant 1)

‘Their privacy is maintained since they are assisted privately when they seek health services. Again, youth have specific time service as priority, 2 p.m. and 7 p.m. in the clinic. The challenge sometimes comes when youth decide to come during school hours for reproductive health services and HIV counselling and testing, and when we advise that they can still do that after school, they accuse nurses of a negative attitude.’ (Participant 6)

#### Subtheme 1.2: Lack of commitment to offer Adolescent and Youth Friendly Services by professionals at multiple levels

Commitment is presented as one of the energising forces for motivation and behaviour. In this study, it emerged that nurses’ commitment was essential in implementing and sustaining the AYFS programme. The following statements from participants support this statement:

‘When I say new young nurses should be invested in the programme, we should also consider an employee’s interest. Otherwise, it is a problem since then no one likes the problem except that it is just a must-do.’ (Participant 6)

‘To make matters worse even my colleagues don’t give me any support instead, when they are seeing patients, and I am busy with the queue, they usually complain that I don’t assist them, but when I am off or on leaving, the youth programme activities to stop since there will be no guider. They only assist those seeking medical attention.’ (Participant 10)

#### Subtheme 1.3: Lack of trained professionals to implement Adolescent and Youth Friendly Services

Participants indicate that there is a scarcity of trained nurses to implement the AYFS programme in the clinics of Kganya local area. This is evident from the following extracts:

‘I think is because of the lack of training. For example, only one nurse in this clinic is trained in Adolescent and Youth Friendly Services. We only implemented the programme according to the policies years ago. Two enrolled assistant nurses were working on the programme, but only one was trained formally, but she has since passed on, and since then, the youth friendly service programme has been on and off.’ (Participant 4)

‘Lack of training of personnel is a problem because we’re only two trained nurses and I feel that we were not well equipped with the skills and knowledge except that we just love the programme and are committed to our work and that we cannot refuse if my managers give us task but it is overwhelming.’ (Participant 7)

#### Subtheme 1.4: Lack of infrastructure and material resources problematic for proper implementation of Adolescent and Youth Friendly Services

This study reveals that a lack of resources is experienced as a major challenge in implementing AYFS. The resources range from human to organisational and materials, as confirmed by the following extracts:

‘The other challenge we face is the lack of educational materials, for example pamphlets, because when the programme was still under love life, pamphlets magazines were available and very helpful to the youth, but there is nothing except mouth-to-ear education.’ (Participant 3)

‘The last challenge I can remember is that the space specifically created for youth services is too small, and there is a shortage of personnel. In my view, if I am responsible for youth, I should specifically be concentrating on the youth. Because in some instances, you will find that I am also called or responsible for doing the other nursing activities at the time for youth service. This has a bad impact since something might happen the minute you are gone.’ (Participant 11)

‘The other challenge is that we lack enough resources for the smooth running of the programme. We don’t have a music system, or a television set which will help the youth will be able to watch educational dramas. Another important challenge we lack is a dedicated space for the youth due to poor structure. These resources I have mentioned make the environment to be conducive to the youth.’ (Participant 9)

#### Subtheme 1.5: Existing poor relationship between nurses and youth that affects the implementation of Adolescent and Youth Friendly Services

If adolescent-related health services are not youth friendly, young people will not use them. Poor relationships between youth and nurses make AYFS programmes difficult for youth to access. The following quotations show what the participants said:

‘Some youth just cannot talk with other people maybe it’s how they were raised. They just adopt how they talk with people at home and talk with nurses that way. As for nurses, the youth believe that the nurses have a negative attitude.’ (Participant 2)

‘That is true, to make matters worse other nurses also have a negative attitude towards attending youth or this programme in general. Do nurses make mistakes by judging youth when they consult with sexually transmitted infections or come for family planning services for example, you will hear a nurse asking a teenager: have you started having sex at this age. This negative attitude makes youth fear coming for a consultation, especially when the problem is sexually related.’ (Participant 4)

#### Subtheme 1.6: Lack of funding to support Adolescent and Youth Friendly Services as a possible contributor to the failure of the Adolescent and Youth Friendly Service programme

The participants in this study shared similar views on the lack of funding for the AYFS programme as a challenge for AYFS uptake. The importance of funding is mainly for the provision of resources. The following participants’ statement confirms this assertion:

‘The other challenge I see is that funding might be a challenge from the top-level since programme like HIV is getting funding from FPD and ANOVA originally from America or USAID COMPANY. I think youth is the future of this country, the government should consider requesting funding from other private organisations.’ (Participant 2)

‘ANOVA since it is a supporting partner should spread their wings and support other programmes such as AYFS and not only focus on HIV and AIDS because even youth are suffering from HIV and AIDS.’ (Participant 7)

#### Subtheme 1.7: Lack of monitoring and evaluation of Adolescent and Youth Friendly Services impacts the failure of the programme

The findings of this study reveal that monitoring and evaluation could improve the quality of health provision of the AYFS. This is reflected in the following extracts:

‘The only people who ask if the programme is functional are the sub-district local area managers during ideal clinic assessment, and I also suspect that it is not because they care, but it is the ideal clinic checklist that is requesting the information.’ (Participant 8)

‘Nobody is reviewing or monitoring and evaluating the programme in question. In some instances, youth programme managers even teach the facility to manage what the programme is all about.’ (Participant 6)

‘The only guidance I have is the policy guideline of 2007 for Adolescent Youth-Friendly Service for South Africa. The worst part is that there is no monitoring and evaluation since I manage the programme since 2016.’ (Participant 7)

#### Subtheme 1.8: Negative attitudes of youth, parents and community members experienced by professional nurses towards Adolescent and Youth Friendly Services

The study findings indicate negative attitudes from different groups: the community, professionals and youth. Here are some of the extracts:

‘Sometimes the youth themselves just have a negative attitude towards the health service rendered in the clinic. They have a negative attitude toward just coming to do activities that do not reward them, unlike when they play snooker or cards, they sometimes have hopes of making money. Sometimes youth just succumb to peer pressure if their peers say the programme or youth-friendly activities are boring, they just follow without their mind.’ (Participant 7)

‘The community at large also has a negative attitude towards this programme since they feel it disturbs their children is education since the schedule for activities is between 2 p.m. to 5 p.m. which was considering that the school will be out by then. But now every parent is focused on their children’s education as it predicts the future, not some activities which they view as a form of the game which they don’t value that child’s education.’ (Participant 5)

### Theme 2: Suggestions to improve Adolescent and Youth Friendly Services

The results of this study indicate that despite the challenges nurses experience in implementing AYFS, most of these challenges could be overcome. Below are six categories that offer insight into how the implementation of AYFS programmes in clinics could be enhanced.

#### Subtheme 2.1: Appointment of relevant health professionals to implement Adolescent and Youth Friendly Services

Participants in this study report that an AYFS focal nurse could be appointed to ensure that the implementation of the programme is successful and that continuous training of nurses should be implemented. The following extracts support this:

‘We need a trained or appointed focal person. It is challenging because the government expects us to do the job but does not train us. We are just given policies to follow but don’t know the practical part of it.’ (Participant 3)

‘It’s important to appoint and train young nurses for the AYFS programme. Additionally, management should support these nurses by engaging with them at the grassroots level. At least two more staff members per facility should be trained to support the focal person.’ (Participant 11)

#### Subtheme 2.2: Training of health professionals to implement Adolescent and Youth Friendly Services

The findings of this study suggest that more nurses should receive training to improve their skills in implementing the AYFS programme:

‘At least two more staff members per facility should be trained to give support to the focal person. Included in the two people, it must be the operational managers. I don’t see the reason why the manager can manage people or supervise what she doesn’t know.’ (Participant 4)

‘The training should be done, and each facility should have at least two or more people trained on the programme to assist each other. This should be done because the trained people’s age be taken into consideration. There should also be an initiative for Adolescent Youth Friendly Programme.’ (Participant 6)

‘I recommend that at least managers be trained on the adolescent youth programme so they can be able to support us. At least two people per facility should be trained on AYFS and should be less than 35 years of age so he can cope with the demand of the youth activities.’ (Participant 11)

#### Subtheme 2.3: Establishment of well-equipped infrastructure dedicated to Adolescent and Youth Friendly Services

Based on the study findings, the participants felt that the AYFS did not work because the building structure was too small to have a designated room for rendering health services to adolescents and youth. The below extracts from participants confirm this:

‘Every clinic should also have a designated room which is specifically for youth activities.’ (Participant 3)

‘The Department of Health should just go away with the small houses they call clinics.’ (Participant 7)

‘The Department should include the AYFS programme in its budget so that it can be supplied with the necessary resources. The clinic’s structures should be improved, or containers are purchased so that the youth can perform or seek health service privately without mixing with other people.’ (Participant 9)

#### Subtheme 2.4: Provision of relevant and enough material resources for easy implementation of Adolescent and Youth Friendly Services

Participants in this study recommend an adequate supply of material resources like educational posters and equipment for effective implementation of the programme as seen in the following extracts:

‘Department of Health should supply us with enough equipment such as music systems and posters.’ (Participant 2)

‘I recommend that every clinic be supplied with IEC [*Information, Education and Communication*] materials or pamphlets.’ (Participant 3)

‘I think, like the manager of the clinic, she needs to shout in case of shortages of learning materials or other resources since she is well connected to the people up there …’ (Participant 10)

#### Subtheme 2.5: Awareness campaigns and programmes about Adolescent and Youth Friendly Services in communities for information giving

The participants suggest starting awareness campaigns and health education efforts to inform the community about the AYFS programme and its importance in providing health care services to adolescents and youth by either having promotion material and pamphlets or awareness campaigns with health education sessions:

‘I recommend that every clinic be supplied with IEC [*Information, Education and Communication*] materials or pamphlets. The pamphlets should also be printed in the language the community understand most.’ (Participant 3)

‘The community should be given health talk about the importance of Adolescent Youth Friendly Services. The clinic structure should also be improved, or a specific area be created for the youth.’ (Participant 5)

‘The community should just take part in building the youth and nurses can promote this by educating adults by setting good parental example.’ (Participant 7)

#### Subtheme 2.6: Applications and requests for funding to support Adolescent and Youth Friendly Services and its sustainability

The study participants emphasise the importance of funding for AYFS and recommend seeking support from non-profit organisations. Here is what two of the participants shared:

‘I think clinics should be supported by these NGOs so that implementation of Youth Friendly Services Programmes is fast-tracked.’ (Participant 3)

‘I suggest that the same effort given to other programmes such as ante-natal is given to youth-friendly services. Also, NGO involvement should be seen through financial support.’ (Participant 10)

## Discussion

The AYFS is an integral programme crafted to draw in and cater for the health requirements of adolescents conveniently and promptly. Furthermore, it is instrumental in retaining young customers for continued sexual and reproductive health services (Vukapi 2020). This study sheds light on the challenges experienced by nurses in implementing the AYFS programme in the clinics of Kganya local area in the Capricorn district, Limpopo province. Furthermore, suggestions to improve AYFS are discussed as the second theme that emerged from this study.

### Challenges experienced related to the implementation of Adolescent and Youth Friendly Services

The first theme that emerged relates to the challenges experienced during the implementation of AYFS. According to the participants in this study, young people have difficulty adhering to the AYFS time schedules in the clinics of Kganya local area. The distance to the clinics also emerged as a challenge. Ninsiima, Chiumia and Ndejjo (2021) also found that the distance to the clinic is one of the factors influencing access to and utilisation of the youth-friendly sexual and reproductive health services in sub-Saharan Africa. Distances to the clinic deprive adolescents and youth of access to health care services and pose a severe threat to the realisation of SGD 3, which strives to ensure healthy lives and well-being for all.

In addition, the lack of commitment of nurses to offer AYFS emerged as a challenge. In line with the current study findings, the study of Awang et al. (2020) reported that the nurses’ commitment is a prerequisite for the effective implementation and provision of AYFS in clinics. Moreover, in this study, a lack of training is seen to be a challenge for nurses in implementing AYFS in the clinics of Kganya local area. The findings of this study are consistent with the systematic review conducted by Akinwale et al. (2022) highlighting the lack of training on AYFS as a challenge among nurses in the health facilities. The training of nurses is vital for expanding and enhancing the AYFS programme and for developing new interventions to promote the well-being of adolescents and youth (James et al. 2018; Thomée et al. 2016).

A lack of infrastructure and material resources emerged as another challenge to proper AYFS implementation. In South Africa, the Ideal Clinic initiative was implemented to ensure that clinics had high-quality infrastructure and resources (NDoH 2017). However, participants in this study report that there is limited infrastructure for the implementation of the AYFS. Moreover, in other South African health sub-districts, studies have found that inadequate infrastructure and weak coordination of resources challenge the implementation of the AYFS programme (James et al. 2018; Nakasone Chimbindi et al. 2020; Van Pinxteren, Cooper & Colvin 2021). Adolescents and young patients are reported to communicate disrespectfully and inappropriately with nurses, which negatively affects the implementation of AYFS. Similarly, the findings of the phenomenological study among community members aged 18 years and above in Pakistan reveal that numerous factors aggravate disrespectful behaviour towards and communication with nurses (Baig et al. 2022). These factors include long waiting times, neglecting adolescents and youth when they come for a consultation, inadequate medical supplies and nurses’ use of mobile phones while on duty. Thus, Thomée and colleagues surmised that nurses previously trained in AYFS exhibit significantly more youth-friendly attitudes than nurses who are not trained (Thomée et al. 2016).

A lack of financial support is another challenge to successfully implementing the AYFS programme. According to the participants in this study, ANOVA and other private organisations should spread their financial wings and support the implementation of the AYFS programme. A study conducted in the Northeastern Peninsular of Malaysia supports the findings of this research, emphasising the importance of financial assistance to ensure the successful implementation of adolescent-friendly health services (Awang et al. 2020). In South Africa, non-governmental organisations are crucial in addressing health system challenges, improving coordination and collaboration, and providing adequate financial and other support to the NDoH (Pillay 2022). Therefore, financial support from several non-governmental organisations for implementing the AYFS programme in the clinics of Kganya local area would be beneficial.

In addition to the challenges experienced by nurses in implementing the AYFS programme in the clinics of Kganya local area, a lack of monitoring and evaluation emerged as a negative factor. According to the participants, operational managers in clinics do not review or monitor how functional the AYFS is. For example, participants report that when the AYFS focal nurses are off duty, no one is delegated to substitute for them. Also, participants report that the functionality of the AYFS programme is only evaluated during Ideal Clinic Assessments by the local area and sub-district managers of Kganya. This is because a fully operational AYFS programme in each clinic is a crucial prerequisite for achieving the status of an Ideal Clinic, as highlighted in Element 44 of the Ideal Clinic Checklist (Ideal Clinic Manual 2020). As other studies have also pointed out, a lack of manager support and inadequate programme evaluation and monitoring hinders the effective implementation of the AYFS programme (Koon, Norris & Goudge 2014; Naicker 2022). Thus, continuous support, evaluation and monitoring from involved stakeholders is necessary for the AYFS programme to achieve its goal of mitigating health challenges faced by adolescents and youth.

The AYFS programme has been vigorously promoted by the NDoH and partners in South Africa to guarantee that the health and well-being of young people aged between 10 and 24 years receive uniform health care standards. Notably, the AYFS programme, as outlined in the 2017 National Adolescent & Youth Health Policy, is a comprehensive and integrated approach aimed at achieving multiple objectives. These objectives include providing sexual, reproductive and ante-natal health services, preventing HIV and AIDS and TB, introducing innovative youth-oriented programmes and technologies and preventing violence and substance abuse (Pillay, Manderson & Mkhwanazi 2020; NDoH 2017). However, this study found that adolescents, youth, parents and the community sometimes hold negative views of the AYFS programme in the Kganya local area clinics. According to the participants, adolescents and youth do not perceive the AYFS programme to be as rewarding as other leisure activities, such as playing snooker or cards, which offer the prospect of making money.

On the other hand, the community also has a negative view of the programme, as they believe it disrupts their children’s education. The findings of this study are supported by a systematic review examining factors affecting adolescent and youth access to, and use of, sexual and reproductive health services in sub-Saharan Africa. The review found that barriers include individual barriers, structural barriers, cultural barriers and socio-economic barriers (Ninsiima et al. 2021). According to the AYFS-trained nurse participants in this study, some adolescents and youth experience individual barriers when it comes to the utilisation of the AYFS in the clinics of Kganya local area. Individual barriers refer to adolescents and youth having incomplete or incorrect knowledge of AYFS, including myths and misconceptions about the health services provided (Ninsiima et al. 2021). In addition, in line with the findings of this study, the literature suggests that cultural barriers include restrictive norms and stigma around adolescent and youth sexuality, inequitable gender norms and discrimination by communities, parents and health care providers (Geary et al. 2014).

### Suggestions to improve Adolescent and Youth Friendly Services

Nurses participating in this study suggest measures and practices that may be used to improve the implementation of the AYFS in the clinics of Kganya local area, Capricorn district, Limpopo province, South Africa. They suggest that a trained focal nurse for AYFS should be appointed. The findings of this study match those of the study conducted by Vukapi (2020), where participants recommend training nurses to provide health services to adolescents and youth. Moreover, in this study, it is suggested that at least two nurses per facility should be trained to support the focal person in implementing the AYFS. These findings resonate with those of the systematic review conducted in sub-Saharan Africa by Ninsiima et al. (2021), which suggests that nurses should receive comprehensive AYFS training to provide quality health services to adolescents and youth.

In addition to the above suggestion, every clinic should also have a designated room, which is specifically for offering AYFS. Literature corroborates the findings of this study; inadequate or substandard infrastructure in the Limpopo province remains a challenge. For example, in Limpopo, a study by Netshisaulu, Malelelo-Ndou and Ramathuba (2019) found that most health establishments were developed by missionaries and that their infrastructure is old and worn. Similarly, Nyelisani, Makhado and Luhalima (2023) postulated that the lack of equipment, inadequate training and limited infrastructure in Limpopo province make it difficult to provide optimal care to critically ill patients. Thus, Muthelo et al. (2023) suggested that the introduction of proper infrastructures in Limpopo province is required to ensure ideal clinical practice. The intervention suggested would enable AYFS to have its required space.

Moreover, participants in this study suggest the provision of an adequate supply of material resources, including information, education and communication materials and music systems, for the smooth implementation and running of the AYFS programme in the Kganya local area clinics. This study’s findings are corroborated by a study conducted in Uganda, which suggests that the Ministry of Health provides adequate equipment and supplies at health facilities to meet the health needs of adolescents and youth (Bukenya et al. 2017). Another suggestion for successfully implementing the AYFS programme is awareness campaigns in communities, for information giving. According to Ninsiima et al. (2021), to promote the utilisation of the AYFS programme effectively, it is imperative to implement successful strategies such as community outreach and engagement, school health education, peer-led instruction, mass media initiatives and participation in recreational and cultural events. These strategies should be prioritised to ensure the successful adoption of the AYFS programme. Lastly, in this study, participants recommend NGOs’ assistance in funding the AYFS programme, as it would accelerate and improve the programme’s implementation in the Kganya local area, Limpopo province. Similarly, the systematic review findings show that more financial support is needed from various stakeholders to implement the AYFS programme successfully and to cater for the health needs of adolescents and youth (Huaynoca et al. 2015; Michaud et al. 2019).

### Limitations of the study

The study was conducted in the clinics of Kganya local area, Capricorn district, Limpopo province, South Africa. However, the findings of this study cannot be generalised to other local areas or districts in this province. Nonetheless, describing the setting and findings in detail may allow other researchers to apply the findings to other settings.

## Conclusion

The results of this study confirm that nurses experience many challenges while implementing AYFS. Some of the noted challenges include a lack of trained staff, unavailability of materials and resources and small spaces in the clinics to provide AYFS in private. Nurses are also concerned about the negative attitudes of adolescents and youth, their parents and the community towards the AYFS programme. The study recommends that at least two nurses per facility be trained to support the focal person in implementing the AYFS. The study further recommends establishing a well-equipped infrastructure dedicated to AYFS. Additional research is recommended to identify activities that would be useful to AYFS programmes and to develop strategies to improve the implementation of AYFS.
